# The Reasonable, the Rational, and the Good: On Folk Theories of Deliberative Judgment

**DOI:** 10.1162/opmi.a.24

**Published:** 2025-08-29

**Authors:** Igor Grossmann, Niyati Kachhiyapatel, Ethan A. Meyers, Hanxiao Zhang, Richard P. Eibach

**Affiliations:** Department of Psychology, University of Waterloo, Waterloo, ON, Canada

**Keywords:** rationality, decision-making, person perception, folk theories, practical wisdom

## Abstract

Judgment is often described in terms of an intuitive (System 1) versus deliberative (System 2) dichotomy, yet sound deliberation itself can take more than one form. Building on philosophical traditions and distinctions in treatment of sound judgment in economics and law, we propose that lay conceptions revolve around two distinct types of deliberate judgment: *rational*, emphasizing rule-based and utility-focused reasoning for well-defined problems, and *reasonable*, prioritizing context-sensitive and socially conscious reasoning for ill-defined problems. Across four studies in English-speaking Western samples (Studies 1–4; *N* = 2,130) and a Mandarin-speaking Chinese sample (Study 4; *N* = 697), participants described their notions of “sound” and “good” judgment, evaluated social scenarios, chose between candidates with distinct judgmental profiles, and categorized non-social objects. Results consistently showed that people view both rationality and reasonableness as common forms of deliberate sound judgment, while treating them as distinct. Participants preferred rational deliberation for algorithmic social roles linked to well-defined tasks and reasonable deliberation for interpretive roles linked to ill-defined tasks. Moreover, framing decisions as rational vs. reasonable influenced whether participants relied on rule-based vs. overall-similarity strategies in classification tasks. These findings suggest that lay understanding of sound judgment does not rely on a single standard of judgmental competence. Instead, people recognize that both rationality and reasonableness are critical for competent deliberation on different types of problems in life.

## INTRODUCTION

Imagine a city official tasked with drafting regulations to address severe housing shortages. She reviews data, runs cost–benefit analyses, and consults urban development models to identify the least biased solution—an approach many would call *rational* because it emphasizes consistency and formal rules. Yet, when residents protest the proposed construction, citing moral and cultural concerns, the same official meets with them, weighs competing values, and adapts her plans to accommodate local realities. In these actions, she exemplifies a more *reasonable* approach, aiming to balance multiple stakeholders’ perspectives rather than follow a strictly rule-based, utility-maximizing strategy.

This contrast between formal, rule-based, algorithmic optimization and a more holistic, socially conscious deliberation crops up across countless real-world decisions. From choosing trustworthy advisors to evaluating policy trade-offs, sound judgment might appear to hinge on logical analysis and bias avoidance in some cases, while requiring a context-sensitive approach that attends to the peculiarities of individual cases in others. These examples highlight different forms of deliberate judgment and suggest that the nature of the decision problem shapes which standard people apply.

### Beyond the Intuitive–Deliberative Dichotomy: Rational vs. Reasonable Deliberation

The idea that more than one standard defines human judgment is hardly novel. Aristotle distinguished theoretical knowledge (*episteme*), aimed at universal truths, from practical wisdom (*phronesis*), concerned with ethical and context-specific considerations (Aristotle, trans. 1984). Renaissance humanist Michel de Montaigne advanced a deliberative experiential ideal, urging reflective engagement with lived particulars over strict rules (de Montaigne, 1580/2007). In contrast, René Descartes upheld systematic deduction and reliance on logical clarity as the gold standard for knowledge (Descartes, 1641/1901). David Hume added yet another voice, portraying much everyday judgment as intuitive, arising automatically from custom and sentiment rather than from either formal logic or reflective experience (Hume, 1748/2007). These diverse perspectives highlight that human judgment cannot be reduced to any single mode, whether analytical, intuitive, or experiential.

Building on these philosophical foundations, psychological scientists have sought to develop empirical models of human judgment and decision-making. One prominent example is the dual-process model, which distinguishes between “System 1” (fast, automatic, affect-driven) and “System 2” (slower, more effortful, rule-based) thinking (Evans & Stanovich, 2013; Kahneman, 2011). While this model has been influential in advancing our understanding of judgment, early accounts often treated deliberation as a unitary phenomenon, emphasizing its role in promoting logical coherence and “rational” decision-making (e.g., Tversky & Kahneman, 1974; cf. Baron, 1993). A range of scholars have since questioned the assumption that all slow reflection forms a single, logically oriented “System 2.” Conceptual critiques point out redundancy in two system labels (Keren & Schul, 2009) and even suggest a continuum model in which the same principles govern both intuitive and deliberate judgments (Kruglanski & Gigerenzer, 2011). Empirically, fast conflict detection studies show that logical monitoring can operate under severe time pressure (Bago & De Neys, 2017; De Neys, 2021), whereas tripartite and motivational models split deliberate thinking into multiple subsystems (Evans & Stanovich, 2013; Melnikoff & Bargh, 2018). These findings suggest that a unitary view of deliberation may not capture the rich insights offered by philosophers such as Aristotle, Montaigne, and Descartes. Recognizing this complexity, other social-cognitive models have proposed that there may be multiple modes of deliberate sound judgment, each guided by a different set of priorities and standards of assessment (e.g., Tetlock, 2002).

The idea that there may be distinct, valid standards of sound deliberation can be illustrated by contrasting judgmental norms in the domains of economics, epistemology, and the law. In neoclassical economic models, an agent is deemed “rational” if they maximize personal utility under consistent preferences (Simon, 1957; Thaler, 2015). Deviations from this ideal, whether stemming from cognitive shortcuts or social preferences, are typically framed as “biases” or “heuristics” that undermine rationality (Tversky & Kahneman, 1974), though some scholars note that choices diverging from strict utility maximization may be purposeful if moral or contextual factors guide behavior (e.g., Hertwig & Gigerenzer, 2011; Schwartz, 2000). Beyond economics, in the field of epistemology, “rationality” may be defined in terms of Bayesian logic, which specifies formal rules for forming and updating beliefs based on the quality of accumulated evidence (Bovens & Hartmann, 2004). As with economic rationality, deviations from the ideal of Bayesian epistemology are typically framed as “biases” or “heuristics” that undermine rationality (Tversky & Kahneman, 1974), though some scholars note that these deviations may reflect adaptive error management (e.g., Haselton & Nettle, 2006). The economic and Bayesian definitions of rationality share an emphasis on standards of judgment that can be represented with well-defined formulae/algorithms. By contrast, legal scholars often invoke a “reasonable person” standard to judge whether actions and outcomes are acceptable, taking into account situational particulars and broad norms (Sibley, 1953; Tobia, 2018). Far from insisting on internal consistency or self-serving optimization, reasonableness in law is oriented toward fairness, norms, and appropriate contextual sensitivity (Jaeger, 2025, for a review). In political philosophy, Rawls (1999) similarly distinguished the “rational,” aimed at maximizing one’s ends, from the “reasonable,” which involves recognizing and coordinating with others’ interests. Thus, it appears that scholars in different fields treat different forms of deliberation as central to sound judgment: While economists and Bayesian epistemologists emphasize *rationality* for utility and consistency, lawyers and political philosophers emphasize *reasonableness* for social norms, moral considerations, and flexible contextual adaptation^1^.

### Lay Beliefs: Emerging Evidence

How do laypeople parse these concepts? Recent scholarship started to examine when people rely on “head” (reason) versus “heart” (intuition) in their judgments (e.g., Hsee et al., 2003), and whether this division captures the chief ways humans conceive of *sound judgment*. For instance, Inbar and colleagues (2010) asked Americans how much intuition or reason should guide decisions across various everyday scenarios (e.g., which apartment to rent, which restaurant entrée to order). They systematically varied features such as the *complexity* or *sequentiality* of a choice—finding that participants deemed deliberation more suitable for problems that appeared objectively evaluable, multi-stepped, or logically precise. By contrast, participants saw intuition as more suitable for subjective contexts (e.g., personal tastes or “gut” feelings). Relatedly, intuition is preferred when decisions are framed as matters of authentic self-expression (Oktar & Lombrozo, 2022)—an idea rooted in the “ethic of authenticity,” which holds that some personal commitments should occasionally outweigh strict rational calculation (Varga & Guignon, 2020). Intuition is also preferred when one believes oneself to be an expert in the decision domain (Pachur & Spaar, 2015). Meanwhile, Hsee and colleagues (2015) introduced *lay rationalism* as an individual difference in how much Americans value “hard” facts and cost–benefit reasoning over feelings, showing that those scoring higher on their Lay Rationalism Scale prefer utilitarian (vs. hedonic) products, donate less in high-emotion charity appeals, and save more money. These findings suggest that lay understanding of being “rational” means minimizing emotional influence, at least in the US (also see Li & Hsee, 2019).

Grossmann and colleagues (2020) built on these lines of inquiry by exploring whether *rationality* and *reasonableness* constitute distinct lay standards of sound judgment, drawing explicitly on ideas from economics (which treats rational decisions as strict preference-maximization) and legal scholarship (which frequently invokes “reasonableness” to incorporate social norms and contextual fairness). This research combined linguistic/cultural analyses with a series of experiments. In the first set of studies the distinction was tested using spontaneous descriptions, social perceptions, and large-scale analyses of cultural products (newspaper articles, soap operas, legal opinions, Google Books in multiple languages). Across several Indo-European languages, findings showed that “rational” was spontaneously tied to *self-focused* or instrumental aims, while “reasonable” highlighted *social* or norm-based considerations. Moreover, laypeople consistently saw “rationality” as preference-maximizing (focusing on logic, personal gain, abstract self-interest), whereas they viewed “reasonableness” as context- and norm-sensitive (highlighting social fairness, contextual adaptation, empathy). In subsequent experiments in Canada, the US, and in Pakistan, participants made decisions in classic economic games, such as the Dictator Game (deciding how to allocate money between oneself and another), the Commons Dilemma (choosing how much to harvest from a shared resource), and the Prisoner’s Dilemma (deciding whether to cooperate or defect). Despite their varying cultural and socioeconomic backgrounds (including Pakistani bankers, street merchants, and North American students) participants consistently described *rational* actions as those aiming to maximize personal gain, and *reasonable* actions as balancing personal benefits with fairness. Across these diverse samples, the “rational” choice was viewed as unconstrained by social norms, whereas the “reasonable” choice was seen as attuned to those norms.

Are these findings from Grossmann et al. (2020) simply another way of invoking the “head vs. heart” divide, where rational means unemotional and reasonable means emotionally guided? Or do lay rationality and reasonableness represent two distinct forms of deliberate judgment? It could be that lay understanding of “rationality” simply refers to dispassionate calculation, while lay “reasonableness” relies on emotion-laden intuition. If this is so, the observation of more cooperative behavior in economic games when asked to act reasonably (vs. rationally) in earlier studies (Grossmann et al., 2020) may reflect intuition-driven choice similar to the results in studies by Inbar and colleagues (2010; also see Rand, 2016). Notably, analyses of response times in past studies revealed no significant differences between rational and reasonable conditions (instructions to consider rational or reasonable approaches to choice in economic games; Grossmann et al., 2020; Studies 5–7), suggesting comparable cognitive engagement with the decision scenarios.

Here, we suggest an alternative interpretation of past scholarship on lay theories of judgment. We propose that lay perceivers believe that *both* rational and reasonable judgments can be thoughtful and deliberate, but emphasize different aims (cf. Rawls, 1999). Building on earlier scholarship (Grossmann et al., 2020), lay rationality may stress internal consistency and maximizing personal payoff, whereas lay reasonableness may be oriented toward integrating multiple stakeholders’ preferences and civic norms. Consequently, stereotypic attributes of rationality may involve self-serving competences, while stereotypical attributes of reasonableness may involve socially-conscious concerns (Grossmann et al., 2020; Study 3). Moreover, informed by examples in the opening paragraph, we propose that the lay distinction between rationality and reasonableness extends beyond the social dimension, with the reasoning process and judgmental styles favoring rule-based processes and analytic reductionism on the one hand, and dialogical processes and context-sensitive holism, on the other hand. In short, folk theories of rational and reasonable deliberation may encapsulate a rich array of stereotypes, foci, judgmental styles and reasoning processes (see Table 1).

**Table 1.** Proposed framework to distinguish folk theories of rational and reasonable deliberate judgment.

	**Rational**	**Reasonable**
Stereotypic attributes	Less sensitive to context/mechanical/consistency-focused	Highly sensitive to context/flexible
Focus	Focuses on commensurable values and seeks to reduce them to a single dimension of valuation	Balances incommensurable values that cannot be reduced to a single dimension of valuation
Judgmental style	Reductionist—i.e., excludes from consideration qualities deemed irrelevant or that cannot be efficiently integrated into categories/rules even at the cost of oversimplification	Holistic—i.e., tries to include as much of the available information as possible even at the cost of precision, clarity, or efficiency
Reasoning process	Deliberative reasoning that uses a logical/syllogistic/rule-based process to proceed in generally linear progression from premises to conclusions	Deliberative reasoning that uses dialogic reasoning that references social conventions/norms and rhetorical strategies such as reasoning by analogy, and narratives
Relevant contexts/domains of application/decision space	Well-defined problems—i.e., closed systems where all relevant variables are fixed and known with clear rules for how to integrate these variables; quality of judgments can be objectively assessed	Ill-defined problems—i.e., open systems where the relevant variables are uncertain and/or unknown and/or one lacks clear rules for how to integrate these variables; quality of judgments must be subjectively assessed

If both rationality and reasonableness represent lay standards of deliberate judgment, a question arises about when people switch between them. Building on prior theoretical work (Grossmann & Eibach, 2024; also see Hammond, 2007; Payne et al., 1993; Simon, 1957), we therefore propose that decision ecology informs preferences for rational vs. reasonable judgment (Table 1). Specifically, lay preferences for rationality may concern well-defined problems where self-focused control, algorithmic rules, and bias reduction drive success. Conversely, lay preferences for reasonableness may concern ill-defined problems that require balancing incommensurable values, social input, and context-sensitive adaptation. The hypotheses about the properties (stereotypic attributes, focus, judgmental styles, and reasoning processes) that lay perceivers will attribute to rationality and reasonableness outlined in Table 1 are based on previous theory (Grossmann et al., 2020) and research (Dutta, 2018; Schraw et al., 1995) describing psychological processes and judgmental approaches that are adaptive for coping with well-defined and ill-defined problems, respectively. In other words, we hypothesize that lay-perceivers’ understanding of the decision ecology of each type of problem informs their ideas about what properties of deliberative reasoning are useful for each type of problem, and we further hypothesize that lay perceivers tend to group those adaptive properties together and associate those groupings with the terms “rationality” and “reasonableness.”

### The Present Research

We conducted a series of five studies to test several key hypotheses based on the framework we outlined in Table 1. First, we hypothesized that laypeople consider rationality and reasonableness to be distinct standards for evaluating the soundness of judgment. In particular, we tested the framework’s claim that these are both deliberative types of judgment but that they differ in some of their core qualities. To test this hypothesis, we explored if people understand rational and reasonable concepts as deliberate and thoughtful (vs. emotional and intuitive) in the context of sound judgment (in Study 1a and good judgment in Study 1b), but they do not use them interchangeably because they recognize that the concepts are aligned with distinct properties corresponding to the distinctions outlined in Table 1. Although lay definitions of rationality and reasonableness have been differentiated in previous research (Grossmann et al., 2020), that research primarily focused on laypeople’s beliefs about rationality and reasonableness in the context of economic choices that pit self-interest against others’ interests. The framework in Table 1 proposes that laypeople draw broader distinctions between rationality and reasonableness that have implications beyond those tested in previous research. To investigate this, Studies 1a and 1b explore lay definitions of these judgmental properties in greater depth and with more robust methods than in previous research.

Second, we hypothesized that people’s preferences for rational versus reasonable agents should shift systematically across a range of social roles that differ in their demands for analytic generalization versus holistic, context-specific considerations. To test this hypothesis, we examined how laypeople apply rationality vs. reasonableness as standards across a broader range of everyday judgments than the economic transactions tested previously (Studies 2–3). We further hypothesized that people will prefer rationality as the standard of judgment in well-defined contexts but prefer reasonableness as the standard of judgment in ill-defined contexts in people’s lives (Study 3). We conjectured that participants would opt for rational deliberation in well-defined contexts because such contexts involve specific problems, and effective solutions in these cases depend on self-directed control, established rules, and the reduction of bias. Conversely, we expected participants to favor reasonable deliberation for ill-defined problems, because such problems require balancing incommensurable values, social input, and contextual adaptation.

Third, we hypothesized that the distinction between rationality and reasonableness would extend even to non-social domains. According to the framework in Table 1, laypeople should associate rationality with a reductionist judgmental style—i.e., reduce judgments to the smallest set of relevant considerations while ignoring, downplaying, or trivializing considerations that are seen as non-essential as “noise.” Conversely, laypeople should associate reasonableness with a holistic judgmental style—i.e., include as much of the “good enough” available information as possible even at the cost of precision, consistency, clarity, or efficiency. To the extent that this is true, then it should lead laypeople to expect that even decisions about non-social problems should be made differently when they apply the rational vs. reasonable standard to those problems. To test this hypothesis, we asked participants to indicate their expectations for how a rational person and a reasonable person would categorize non-social objects: by matching a target object by a single feature shared among all objects (a reductionist option) or by focusing on the “good enough” overall similarity even if none of the features were consistently shared among all objects (a holistic option; Studies 4–5)^2^. Assessing whether laypeople extend the distinctions between rationality and reasonableness even to a relatively unfamiliar, non-social task is a strong test of our hypothesis that laypeople recognize these as qualitatively different standards for judgment.

### Transparency and Openness

For each study we report how we determined our sample size, data exclusions, all manipulations and measures (https://osf.io/excny/). Study 1 was exploratory and not preregistered. We preregistered the hypotheses, design, and analyses of Study 2 (https://osf.io/fw2ht), Study 3 (https://osf.io/x54j8/), Study 4 (https://osf.io/7x8zk), and Study 5 (https://osf.io/92vpx). All analyses were performed in *R Statistical Software* (v4.2.3; R Core Team, 2023).

## STUDY 1

We started our research by exploring whether people perceive reasonable persons to be more emotional and intuition-driven than rational persons, or whether both concepts reflect chiefly deliberate qualities. Study 1a examined the link between general perceptions of sound judgment and the attributed characteristics of rationality and reasonableness, while Study 1b focused on good judgment when working through a challenging situation, to zero-in on contexts in which judgmental competence is usually required^3^. In both versions of the study, we took a multi-pronged approach. First, people spontaneously described characteristics of a person showing sound/good judgment. Second, they reported the extent to which they ascribed analytical, moral, and social characteristics to rational and reasonable persons. Methodologically, we relied on natural language processing of word co-occurrences and psychometric factor analyses to examine the semantic understanding of each concept.

If our deliberation account of lay reasonableness and rationality corresponds to folk theories of judgment (Table 1), it will impact attributes of judgmental competence people assign to each concept, revealing distinct semantic and psychometric structures of characteristics associated with each. Specifically, we expected that analytic strengths would be attributed to both, but interpersonal strengths would be uniquely attributed to reasonable people, consistent with prior research (Grossmann et al., 2020). Furthermore, if lay rationality is understood as reductionist judgment in service of a single favored value (and filtering less favored values), psychometric analyses will show distinct clusters of traits attributed to rational people (e.g., greater differentiation between analytical and moral qualities or between agency and communion-oriented characteristics). Conversely, if lay reasonableness is understood as integration-based judgment in service of a plurality of relevant values, the lay concept of reasonableness will show more interconnectedness between traits attributed to reasonable people.

### Methods

#### Participants.

All studies were approved by the University of Waterloo Office of Research Ethics (Protocols 30580 and 40360). We obtained informed consent from all participants. We recruited participants via Amazon’s Mechanical Turk (mTurk) through CloudResearch (Litman et al., 2017) to complete a 5-min survey on the Qualtrics platform in exchange for $0.50. To be eligible for participation, individuals were required to be at least 18 years of age, reside in Canada or the US and possess a HIT approval rating at or above 90%. Study 1a participants were prevented from signing up for Study 1b.

Estimating sample size for advanced psychometric models (up to 30 parameters in Study 1a) and natural language processing studies is complex. Given the exploratory nature of Study 1a, we aimed to ensure content-rich and diverse open-ended content. Therefore, we relied on general rules of thumb rather than formal sample size calculation. To this end, we followed Harrell’s (2015) general guideline of 10–20 observations per parameter in multivariate analyses, while considering Epskamp and colleagues’ (2018) general recommendation for larger samples in psychometric models. Targeting 450 mTurk participants in Study 1a, we oversampled to about 600 (33% increase) to account for expected attrition in mTurk studies (Aguinis et al., 2021). In Study 1b, we mirrored this approach, aiming for an identical sample size. We reviewed open-ended responses to questions concerning definition of sound/good judgment and excluded non-compliant entries (e.g., non-sense, bot-like responses like “VERY NICE”), and those who did not complete the attributes survey. We also included partially completed responses of participants who completed the survey, but possibly stopped before completing the demographics, for our analyses. Table 2 presents the demographic information.

**Table 2.** Demographics of participants in Studies 1–5.

	**Study 1a**	**Study 1b**	**Study 2**	**Study 3**	**Study 4**	**Study 5**
Recruited *N*	635	608	361	509	599	797
Final *N*	465	490	284	464	411	697
Age_mean_ (*SD*)	36.01 (*11.53*)	34.72 (*11.84*)	36.65 (*11.57*)	35.79 (12.63)	25–34^a^	27.24 (*6.94*)
Sex (% female/male/other)	58.1/40.8/1.1	61.6/37.6/0.8	47.3/52.3/0.3	49.1/50/0.9	54.4/45.3/0	60.0/40.0/0
Ethnicity (%)						
Asian-American	6.0	7.8	6.7	^b^	4.9	
African-American	10.5	11.1	20.1		9.6	
White	72.3	71.5	63.0		72.9	
Hispanic	5.3	5.5	6.0		5.6	
Other	5.9	4.1	4.2		7.0	
Income_median_ (US dollars)	50,001–75,000	50,001–75,000	50,001–75,000	50,001–75,000	N/A	
Education (%) / *M* (*SD*)^c^						11.83 (*3.15*)
Less than High School	0.9	0.6	0.1	0.9	0.9	
High School	7.7	10.2	8.1	14.4	11.0	
Some College	35.7	40.5	25.4	28.5	25.0	
College	40.2	32.9	47.7	43.5	47.0	
Post-graduate	15.5	15.8	18.7	12.7	15.3	

^a^ Participants in Study 4 indicated what age group they belonged to (18–24, 25–34, 35–44, 45–54, 55–64, 65–74, 75–84) rather than providing exact age. The estimate reflects the median age group in the sample.

^b^ We did not include ethnicity in Study 3, because we targeted people from Canada (44.1%), the US (29.4%), and the UK (26.5%), with varied ethnicity nomenclature across these countries.

^c^ Chinese participants in Study 5 reported number of years of secondary education.

#### Procedure.

In Study 1a, participants were asked to list five adjectives, in order of importance, which they believed described “a person who shows sound judgment,” starting with the most important. Next, they provided a brief open-ended response to describe what *sound judgment* means to them in their own words (explicitly instructed not to copy definitions from websites/dictionaries). Next, participants were asked to sequentially evaluate three agents (a person who exhibits sound judgment, a rational person, a reasonable person) on a list of 28 characteristics provided (see Table S3 and Figures 2 and S3), from the perspective of how society in general views them; the latter phrase was chosen to ensure we target perception of descriptive norms rather than idiosyncratic personal preferences. The ratings for the person who exhibits sound judgment were always elicited first, while ratings for rational and reasonable persons were presented in a counterbalanced order. Participants were also asked to recall and explain the first task they completed in the study as an attention and data quality check. Finally, participants responded to some demographic questions and the study concluded.

Study 1b followed a nearly identical procedure with three modifications. First, participants listed adjectives and rated an agent who shows *good judgment* in a challenging situation and described what good judgment in a challenging situation means to them. Second, the agents were rated on common attributes of judgmental competence (see Figure 2 for the characteristics), representing agency (i.e., “getting ahead”) and communion (i.e., “getting along”). Due to a programming error, all participants judged the rational agent last after judging the reasonable agent and the good judgment agent in a random order. Notably, this randomization allowed testing if perceptions of reasonable and rational agents vary when evaluated consecutively versus separated by judgment of a “good person.”

#### Materials.

In Study 1a, we chose 28 characteristics (along with ‘rational’ and ‘reasonable’ for evaluation of a person showing sound judgment) based on a pilot test where participants described individuals demonstrating sound judgment, and prior research on person and mind perception (Abele et al., 2021; Gray et al., 2007; Wojciszke et al., 1998; see Table S1 in the online supplement). Participants rated each characteristic on a 7-point scale (1 = *Not at all*, to 7 = *Extremely*). We incorporated a diverse set of analytical and interpersonal characteristics. To reduce the number of comparisons, in main analyses we followed the psychometric model specifications outlined below to select a subset of 11 items making up three factors for main manuscript analyses (see Figure 2): Analytical Reasoning (analytical, logical, objective, smart; *α*_rational_ = .83; *α*_reasonable_ = .87), Empathy & Morality (kind, empathetic, caring, moral; *α*_rational_ = .90; *α*_reasonable_ = .89) and Inner Fortitude (strong, courageous, brave; *α*_rational_ = .88; *α*_reasonable_ = .88). Analyses on other analytical and interpersonal items were largely consistent with those reported in psychometric analyses below (see Figure 2).

In Study 1b, we adapted a set of 9 attributes representing Agency (competent, confident, independent, competitive, intelligent) and Communion (tolerant, warm, good-natured, sincere) from Fiske et al. (2002), along with ‘trusting’ as an additional communion item to ensure equal set of items for each dimension (Agency: *α*_rational_ = .73; *α*_reasonable_ = .70; Communion: *α*_rational_ = .82; *α*_reasonable_ = .77). As in part person perception research, participants rated each characteristic on a 5-point scale (1 = *Not at all*, to 5 = *Extremely*). We also assessed ‘selfish,’ to replicate the differential attributions of self-interest in folk theories of rationality and reasonableness (Grossmann et al., 2020).

#### Natural Language Preprocessing.

After spell check, we excluded responses that did not answer questions (e.g., wrote a paragraph of text when explicitly asked to write adjectives only). For adjective-based analyses, we removed superfluous spaces. This way, we ensured a standardized set of descriptive terms. For adjective-based analyses, we constructed a Document-Feature Matrix (DFM) from the *tidytext* package (Silge & Robinson, 2016) and applied correlation-based similarity metrics to test for similarity of associations of adjectives with ‘rational’ and ‘reasonable,’ respectively. Furthermore, we created the frequency matrix of pairwise co-occurrences and adjusted for relative frequency of each term (A & B co-occurrence / Σ (A, B) frequencies). We treated these correlation- and cooccurrence frequency-based indices as markers of semantic meaning revealed in conjunction with each term, statistically evaluating the difference in distributions of key-term-specific correlations and frequencies.

For narrative responses of the personal understanding of “sound judgment” (Study 1a) / “good judgment in a challenging situation” (Study 1b), we tokenized the text, excluding numeric words, typical English stopwords obtained from the *tidytext* package, and custom stopwords reflecting the restatement of the question (e.g., ‘sound,’ ‘judgment,’ ‘means’) and common trivial opening phrases identified upon initial inspection of the texts (e.g., ‘ability’ and ‘decision’ in “it is the ability to make good decisions”).

#### Psychometric Modelling.

Our first hypothesis was that laypeople mentally differentiate the concepts of *rational person* and *reasonable person*. To test this, we needed to know (a) how many latent trait dimensions organise ratings of each target and (b) whether the same or different dimensions underlie the two targets. We therefore treated the trait ratings as manifest indicators, ran an Exploratory Factor Analysis to uncover the minimal factor structure that accounts for the data, and then used multigroup Confirmatory Factor Analysis (CFA) to test measurement invariance across targets. If forcing identical loadings/intercepts degrades model fit, we can conclude that laypeople rely on *distinct* latent dimensions when thinking about rational versus reasonable agents.

To compare *model fit* of characteristics attributed to rational and reasonable persons, in Study 1a we first identified the number of factors that best explain the variance in the data. Preliminary Principal Component and Exploratory Factor Analyses (EFA) suggested a three-factor solution (per eigenvalues > 1, scree plot results, parallel analysis). We subsequently evaluated an EFA solution with oblique rotation, to allow for correlation between the 3 factors, in *lavaan*. We inspected the standardized loadings of each item on respective factors, seeking to identify items with highest loading and minimal cross-loading (i.e., unique loadings *β* > .350 on a particular factor, and Δ in *β*s > .350), evaluating subsequent confirmatory model fit. In evaluating model fit for the structural equation models analyzed, we employed several indices: the Comparative Fit Index (CFI) where values above .95 suggest excellent fit and values above .90 indicate an acceptable fit, the Root Mean Square Error of Approximation (RMSEA) with values below .06 indicating good fit and values below .10 indicating an acceptable fit, the Standardized Root Mean Square Residual (SRMR) with values less than .08 reflecting acceptable fit, and the Goodness of Fit Index (GFI) where values above .90 denote satisfactory fit (Hu & Bentler, 1999), thus providing a comprehensive assessment of model adequacy. We selected top four items loading on the first factor (analytical, logical, objective, smart) and the second factor (kind, empathetic, caring, moral) and top three items uniquely loading on the last factor (strong, courageous, brave), resulting in a model with excellent fit, CFI = .960, RMSEA = .081, SRMR = .059, GFI = .946.

Applying the same criteria in Study 1b (eigenvalues > 1, scree plot results, parallel analysis), a two-factor model described the data best, consistent with prior work on Agency and Communion (Bakan, 1966; Wiggins, 1991). The confirmatory 2-factor model with five items each loading on the first factor (intelligent, confident, competent, independent, competitive) and the second factor (good-natured, warm, tolerant, sincere, trusting) showed an acceptable fit, CFI = .936, RMSEA = .076, SRMR = .055, GFI = .955.

To test for *measurement invariance* of rational and reasonable judgments, in both studies we formally evaluated the similarity of ratings of characteristics for rational and reasonable persons. We contrasted a configural model where factor loadings and intercepts were free to vary, with a constrained model, where factor loadings and intercepts were set to be equal across the rational and reasonable groups. The comparison of these models provided insights into the degree of measurement invariance: if the constrained model did not significantly worsen the fit, it would suggest metric invariance (equivalence in factor loadings) and scalar invariance (equivalence in item intercepts).

### Results

#### Are Reasonable Persons Perceived as Less Deliberate Compared to Rational Persons?

**Figure 1.**
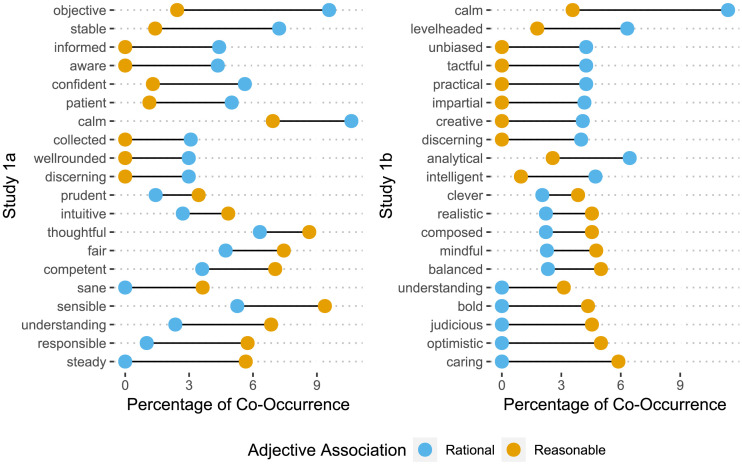
Top ten most frequent co-occurrence of Adjectives with ‘rational’ (blue) and ‘reasonable’ (orange) when asked to describe most important characteristics of a person showing sound judgment (Study 1a) / good judgment in a challenging situation (Study 1b). Adjectives are ordered from those most associated with ‘rational’ to those with ‘reasonable.’ Dumbbell nodes represent the percentage of each adjective’s co-occurrence relative to the sum of independent occurrences of each pair of terms.

We examined adjective co-occurrences to assess semantic similarity of ‘rational’ and ‘reasonable’ concepts to deliberate vs. intuitive characteristics in participants’ descriptions of a person showing sound judgment (Study 1a) / good judgment in a challenging situation (Study 1b). In both versions of Study 1, participants chiefly relied on deliberate qualities such as ‘thoughtful,’ ‘level-headed,’ ‘attentive,’ or ‘discerning’ (Figure 1, also see Tables S2 and S4). Both ‘reasonable’ and ‘rational’ were more likely to be associated with ‘thoughtful’ than ‘intuitive,’ with notably greater co-occurrence of ‘thoughtful’ with ‘reasonable’ rather than ‘rational’ (Figure 1). Similar results emerged in supplementary analyses (Figures S1 and S4), when estimating co-occurrences via the positive Pointwise Mutual Information to account for the differential frequency of individual terms in the corpus. Overall, these analyses showed little evidence that reasonable persons were perceived as less deliberate compared to rational persons.

#### Rational Objectivity vs. Reasonable Understanding.

In both versions of Study 1, the Wilcoxon Signed Rank Test across all pairwise correlations of adjectives in a dataset revealed a statistically significant difference in the patterns of associations with ‘rational’ as compared to ‘reasonable,’ Study 1a: *V* = 24,971, *p* < .001; Study 1b: *V* = 21,273, *p* < .001. As Figure 1 demonstrates, the types of deliberate qualities participants associated with the rational vs. reasonable concepts varied: ‘rational’ was more likely to co-occur with adjectives representing algorithmic qualities—i.e., ‘objective,’ ‘informed,’ and ‘discerning’ (Study 1a)^4^ / ‘analytical,’ ‘unbiased,’ and ‘impartial’ (Study 1b), whereas ‘reasonable’ was more likely to co-occur with adjectives representing holistic, social qualities—i.e., ‘sensible,’ ‘understanding,’ and ‘thoughtful’ (Study 1a) / ‘caring,’ ‘judicious,’ and ‘balanced’ (Study 1b)^5^.

#### How Common Are Reasonable and Rational Concepts in Descriptions of Sound/Good Judgment?

Across 429 unique adjectives in Study 1a (total *n* = 2,320 entries), most common terms concerned ‘intelligent,’ ‘smart,’ ‘thoughtful,’ and ‘wise,’ followed by ‘rational.’ Further, top ten terms included ‘trustworthy,’ ‘logical,’ ‘honest,’ ‘calm,’ and ‘reasonable.’ ‘Rational’ and ‘reasonable’ each appeared more often than characteristics such as ‘fair,’ ‘reliable,’ ‘objective,’ and ‘practical.’ Across five adjectives participants provided, ‘rational’ was the fifth most mentioned (61 times), showing more than 10 times the expected probability of mentioning (probability of mention = .026, 95% CI [.020, .034]), significantly higher than the expected probability of .002, *p* < .001 (see Table S2 in the online supplement). Likewise, ‘reasonable’ was tenth most mentioned (49 times), also demonstrating an effect size that was more than 10 times the expected probability of mentioning (probability of mention = .021, 95% CI [.016, .028]), well above the expected probability of .002, *p* < .001.

Similarly, across 453 unique adjectives Study 1b participants used to describe a person showing good judgment in a challenging situation (total *n* = 2,444 entries), top ten most common terms were ‘calm,’ ‘smart,’ ‘intelligent,’ ‘thoughtful,’ ‘decisive,’ ‘wise,’ ‘honest,’ ‘patient,’ ‘fair,’ and ‘level-headed.’ ‘Rational’ and ‘reasonable’ were again significantly more frequent than most other terms, appearing more often than characteristics such as ‘ethical,’ ‘sensible,’ ‘considerate,’ and ‘flexible.’ Across five adjectives participants provided, ‘rational’ was the 11^th^ most mentioned (39 times; probability of mention = .016, 95% CI [.011, .022]), significantly higher than the expected probability of .002, *p* < .001. Likewise, ‘reasonable’ was 30^th^ most mentioned (16 times; probability of mention = .007, 95% CI [.004, .011]), well above the expected frequency, *p* < .001^6^. Overall, participants spontaneously used ‘rational’ and ‘reasonable’ to describe distinct features of a person demonstrating sound / good judgment, and they did so at the rate well above what would have been expected by chance.

#### Trait-Ascriptions to Rational and Reasonable Persons in Study 1a.

**Figure 2.**
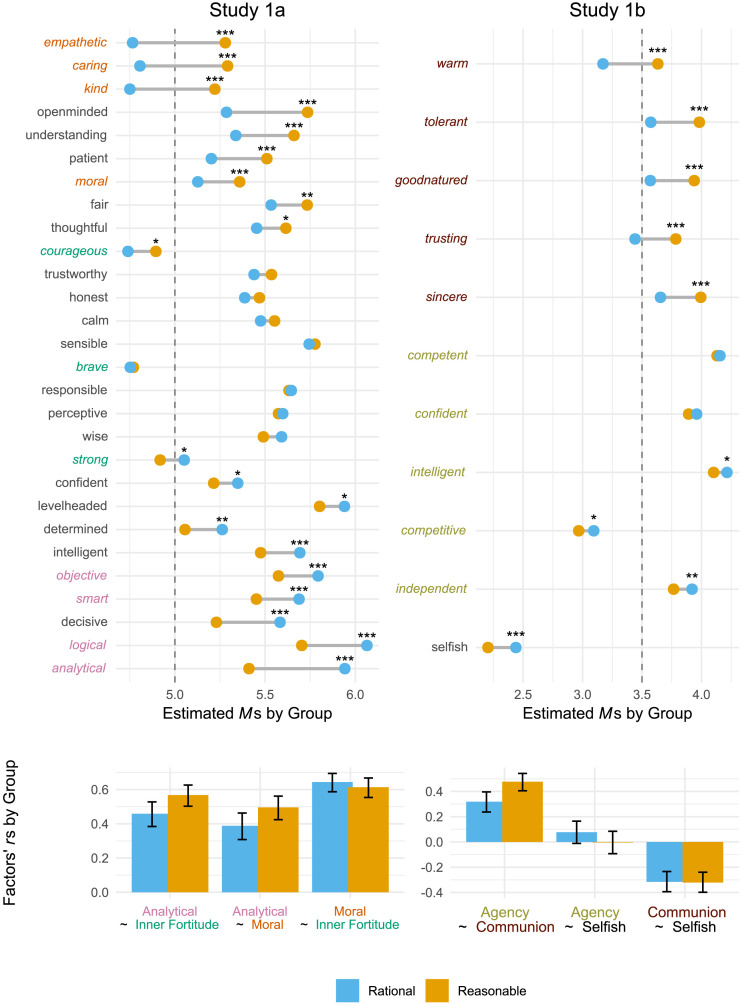
Qualities attributed to rational and reasonable persons in Study 1. Color-coded adjectives reflect Analytical, Moral, and Inner Fortitude items (Study 1a)/Agency and Communion factors (Study 1b). Top panels: Estimates from linear mixed model with responses to all characteristics nested in participants, with target order (rational vs. reasonable) as a covariate and false discovery rate correction for multiple testing. Dashed vertical line delineates effects 1 unit above midpoint of the 1–7 scale in Study 1a / half a unit above the midpoint of the 1–5 scale in Study 1b. Bottom panels: Pearson’s correlations and 95% CIs of average scores across items making up each factor. ***p < .001, **p < .01, *p < .05.

In the psychometric analyses of trait-ascriptions to rational and reasonable persons, we formally evaluated the comparability of the measurement models for rational vs. reasonable persons. In Study 1a, we compared the fit of the 3-factor measurement model of Analytical, Moral and Inner Fortitude latent trait-ascription factors participants relied on when evaluating rational and reasonable persons (see supplementary exploratory factor analyses indicated the best fit of a 3-factor solution; see items loading on each factor in Figure 2). Testing model fit revealed that a metrically invariant model—i.e., constraining loadings to be equal across ratings of rational and reasonable persons—did not result in a significant worsening of fit compared to unconstrained configural model, Δ*χ*^2^(8) = 11.864, *p* = .157. However, moving to the scalar invariant model (constraining both loadings and intercepts) revealed a significantly worse fit, Δ*χ*^2^(8) = 47.743, *p* < .001, indicating non-equivalence of item intercepts between groups. In other words, participants showed non-negligible differences in the conceptualization of Analytical, Moral an Inner Fortitude factors for rational as compared to reasonable persons, with the same score on a particular factor might represent different levels of that factor in the rational group compared to the reasonable group.

Given evidence of a metric but not a scalar measurement invariance across rational versus reasonable targets in Study 1a, we focused on item- (rather than factor-) level comparisons of means in Figure 2. The dumbbell plot on the top showcases the estimated means for trait-ascriptions by target group (also see Figure S3 and Table S3 for the 95% CI around the estimates). Because we observed a halo effect for both targets (ratings for all items were on average above scale midpoint; see top left of Figure 2), we used a somewhat more stringent criterion—one unit above scale midpoint—to evaluate our hypothesis concerning ascription of deliberative and interpersonal characteristics to rational and reasonable persons. Consistent with the open-ended results above, participants ascribed deliberate traits to both targets, with characteristics such as ‘analytical,’ ‘level-headed,’ ‘open-minded,’ ‘understanding,’ ‘thoughtful’ and ‘patient’ all rated at least one unit above scale midpoint (dashed vertical line in the dumbbell graph on the top left of Figure 2 and in Figure S3). Also consistent with the open-ended responses, we observed nuanced differences in the type of deliberate qualities participants ascribed to rational as compared to reasonable persons. Rational persons were relatively more analytical, logical, and objective than reasonable persons. Conversely, reasonable persons were relatively more likely open-minded, understanding, patient, or thoughtful than rational persons.

Replicating Grossmann et al. (2020), interpersonal characteristics such as ‘kind,’ ‘caring,’ or ‘empathetic’ were uniquely attributed to reasonable persons—respective estimates were to the right of the vertical dashed line in Figure 2 for reasonable persons, yet to the left of this dashed line for rational persons. Moreover, reasonable persons were attributed significantly higher interpersonal characteristics than rational persons for most interpersonal characteristics we tested—‘empathetic,’ ‘caring,’ ‘kind,’ ‘open-minded,’ ‘understanding,’ ‘patient,’ ‘moral,’ ‘fair,’ ‘thoughtful’ (Figure 2 and Figure S3), while we did not observe significant differences for such traits as ‘wise,’ ‘perceptive,’ ‘responsible,’ ‘brave,’ ‘sensitive,’ ‘calm,’ ‘honest’ or ‘trustworthy.’

If folk theories of reasonableness concern holistic judgment accommodating a plurality of relevant values, then perceivers should expect reasonable persons to integrate analytical and socially conscious characteristics more extensively than rational persons^7^. To test our prediction concerning greater interconnectedness of traits ascribed to reasonable vs. rational persons, we estimated Pearson’s correlation coefficients of average scores across items making up Analytical, Moral, and Inner Fortitude factors (recall, metric invariance allowed for comparisons of factor loadings; we relied on average scores across items to estimate 95% CIs, with CFA-based factor loading correlations yielding a very similar picture). Consistent with our predictions, when participants evaluated reasonable (vs. rational) persons, ratings on Analytical traits were significantly more likely to converge with ratings on Moral and Inner Fortitude traits: *t*_moral_(497.14) = 3.24, *p* < .001; *t*_fortitude_(498.85) = 3.60, *p* < .001 (bottom left of Figure 2). We observed no significant differences between target-specific associations between Moral and Inner Fortitude traits, *t*(437.13) = 1.67, *p* = .096. In other words, Study 1a participants perceived more interconnectedness between analytical and social-moral traits for the lay concept of reasonableness compared to the lay concept of rationality.

#### Trait-Ascriptions to Rational and Reasonable Persons in Study 1b.

Comparison of measurement models of Agency- and Communion-related attributions to rational and reasonable persons revealed a significant worse fit of the metrically invariant model, in which we constrained factor loadings across rational and reasonable groups, compared to the unconstrained configural model, Δ*χ*^2^(8) = 15.847, *p* = .045, indicating significant structural differences in Agency and Communion factors when evaluating rational and reasonable persons. Following the Study 1a analytical plan, we therefore chiefly focused on item-level analyses.

Like in Study 1a, we observed a halo effect for both targets—all positively loaded items were rated above scale midpoint; see top right graph in Figure 2). Study 1b relied on a 5-point scale, thus we adopted a somewhat more stringent criterion of half-a-unit above scale midpoint to evaluate our hypothesis concerning ascription of interpersonal characteristics to reasonable versus rational persons. The top right graph in Figure 2 indicates that agentic qualities were attributed to both targets, except for ‘competitive’ (also see Table S5 for 95% CIs), with average ratings on all other agentic traits to the right of the vertical dashed line. Additionally, participants rated rational persons relatively more ‘independent’ and ‘intelligent’ than reasonable persons, and rated reasonable persons as relatively more communal on each item than rational persons. Moreover, while participants perceived both rational and reasonable persons as non-selfish, this perception was even stronger for reasonable persons, who were rated as significantly less selfish than their rational counterparts (Figure 2), conceptually replicating earlier work (Grossmann et al., 2020).

Following Study 1a method for evaluating interconnectedness of traits ascribed to reasonable vs. rational persons, we estimated Pearson’s correlation coefficients of average scores across items making up Agentic and Communal factors, along with the Selfishness item. Consistent with our predictions and Study 1a results, when participants evaluated reasonable (vs. rational) persons, ratings on Agentic traits were significantly more likely to converge with ratings on Communal traits: *t*(586.56) = 2.89, *p* = .004 (bottom right of Figure 2). We observed no significant differences between target-specific associations between ascription of selfishness and either Agentic or Communal traits, −.05 < *t*s < 1.05 = 1.67, *p*s ≥ .294. In other words, Study 1b participants perceived more interconnectedness between agentic and social traits for the lay concept of reasonableness compared to the lay concept of rationality.

Overall, Study 1 demonstrated that laypeople typically associate deliberate qualities with both rational and reasonable persons. Furthermore, when laypeople define sound and good judgment both rational and reasonable qualities frequently come to mind. People perceived both concepts as deliberate and agentic rather than intuitive, yet salient features of rational persons appeared more likely to concern bias reduction and logical analysis, whereas salient features of reasonable persons appeared to focus more on socially conscious, interpersonally sensitive characteristics. Moreover, people were more likely to integrate analytical and socially conscious characteristics when evaluating reasonable as compared to rational persons, suggesting that folk theories of reasonableness (vs. rationality) are more likely to concern holistic judgment accommodating a plurality of social and analytical judgmental competencies. Given these nuances in semantic understanding of lay rationality and reasonableness, in the next study we sought to explore how people apply these folk concepts when evaluating and selecting for distinct social roles where sound judgment is needed.

## STUDY 2

**Figure 3.**
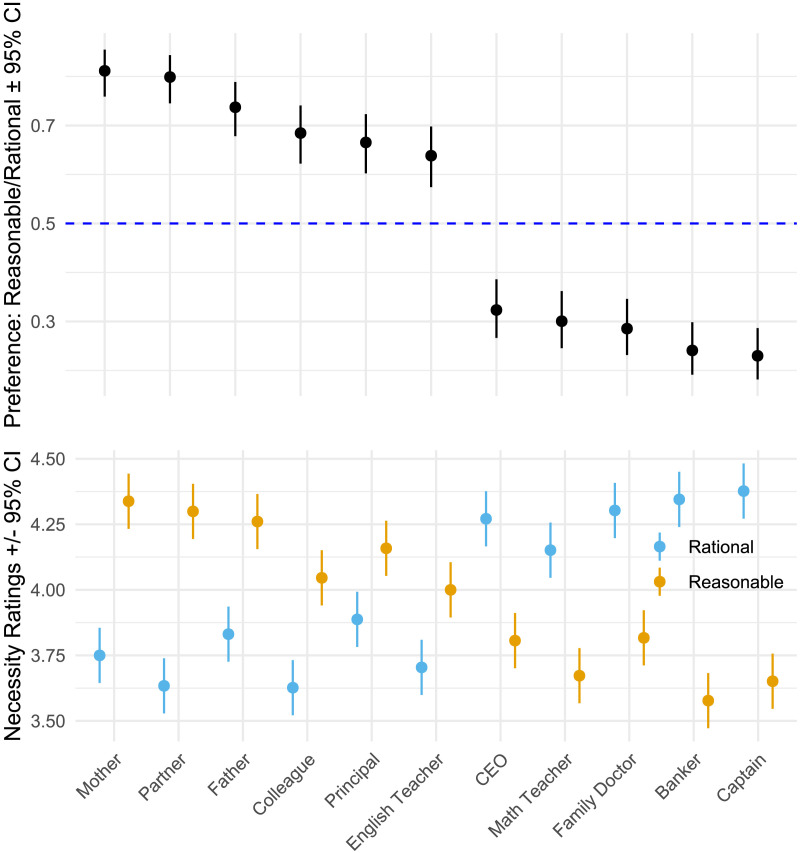
Top Panel: Preference proportions for reasonable vs. rational agents in social roles. Displays proportions (with 95% CI) derived from logits in generalized mixed models. The dashed line at .50 indicates parity; above this, preference leans towards reasonable agents, and below, towards rational agents. Bottom Panel: Necessity ratings for rationality and reasonableness by role (1–5 rating scale). Shows estimated means and 95% CI. Ratings indicate moderate to high necessity (3–4) for both rationality and reasonableness across rule-based (on the right) and holistic roles (on the left).

When facing challenging decisions in everyday life, sometimes it may be of benefit to apply standardized rules, while other times sound judgment may call for holistic consideration of the social context and interpersonal relationships. Do preferences for rational versus reasonable agents shift systematically across a range of social roles that differ in their demands for analytic generalization versus holistic, context-specific considerations? In Study 2, we assessed preferences for rational or reasonable agents across social roles where sound judgment is needed, selecting roles that a priori emphasize analytic judgment that involves general rules or formulas (e.g., math teacher) or holistic judgment that involves context-specific interpretive work (e.g., English teacher, see Figure 3). We hypothesized that laypeople would prioritize rationality for roles, such as a math teacher, which involve tackling well-defined problems that can typically be solved using algorithmic, rule-based judgment, whereas they would prioritize reasonableness for roles, such as a school principal, that involve tackling ill-defined problems where solutions require context-sensitive balancing.

We preregistered that people will be more likely to favor rational agents for positions requiring an algorithmic, rule-like judgment (e.g., CEO of a large company, investment banker, army captain, math teacher) and more likely to favor reasonable agents for positions requiring holistic, context-specific interpretation (e.g., parents, English teacher, school principal, romantic partner, colleague at work). Per pre-registered plan, we probed the preferences for rational and reasonable agents across different social roles by using both binary choices (pre-registered hypothesis 1) and rating-scale-based responses assessing relative preference for rational and reasonable agent for each role (pre-registered hypothesis 2). Moreover, because different roles may differentially favor salience of social and algorithmic considerations, we evaluated the extent to which appraisals of these roles in terms of different interpersonal (warmth, expected interaction frequency, trust) and algorithmic considerations (logic, algorithmic) statistically account for rational vs. reasonable actor preferences.

### Methods

#### Participants.

MTurk participants completed the study on the Qualtrics platform. Participants were offered USD $2.15 to complete the 20-minute survey. To be eligible for participation, individuals were required to be at least 18 years of age, reside in one of Canada, the U.S., the United Kingdom, or Ireland and possess a HIT approval rating at or above 90%. Screening non-compliance per pre-registered criteria (e.g., incomprehensible response, copy/paste survey question in open-ended response field) and in line with the pre-registered power analysis, the final sample consisted of 284 participants (Table 2). We powered for effect size *r* = .21 (based on estimates of average effect size in social psychology; Richard et al., 2003). Per G*Power 3.1.9.2, with alpha and beta each set to 5%, we would require 284 participants to conduct a two-tailed test.

#### Procedure.

Participants first completed the binary choice task where they were asked to choose between a rational or reasonable person for eleven social roles: math teacher, army captain, banker, CEO, family doctor, English teacher, school principal, father, mother, romantic partner, and colleague. We selected these roles to capture a wide breadth of targets one may typically turn to when seeking sound judgment, while varying their relative social status, typical masculinity/femininity, and age. We pre-registered classification of these roles based on their common characterization as signalling logical, rule-based qualities on the one hand, as well as interpersonally sensitive, holistic qualities, on the other hand (also see Supplementary Figures S6–S7 for confirmatory results of participants’ ratings of each role on the logical/rule-based and interpersonal evaluative dimensions).

Next, participants rated how necessary it was for a person in each of the eleven social roles to be rational and to be reasonable on 1 (“not at all”) to 5 (“a great deal”) scales. Each role was evaluated sequentially in a random order. Participants also rated the similarity of each social role (e.g., “Rate the similarity between a typical math teacher and a typical CEO of a large company”) on a 1 (“not at all similar”) to 9 (“extremely similar”) scale. These judgments were divided into four blocks of questions with presentation order randomized (see online supplement for exploratory analyses of similarity judgments; Figure S5). Participants also provided their impressions of a typical person in that role on interpersonal characteristics (e.g., “How warm is a math teacher toward other people?;” other characteristics included trust, interpersonal closeness, interaction frequency) and rule-based characteristics (e.g., How logical is a math teacher?”; other characteristics included logic, algorithmic, pragmatism) on a 1 (“not at all”) to 5 (“extremely”) scale. As a data quality check, participants recalled and explained the main task they completed in the study. Finally, they provided their demographic information.

### Results

Following pre-registration, we first estimated preferences for rational over reasonable agents for a given social role in a generalized linear mixed model with forced-choice preferences (chosen = 1/not chosen = 0) nested within participants and a dummy-coded classification for social roles (analytic = 1/holistic = 0). Estimates and 95% confidence bands (top of Figure 3) indicate that participants were significantly more likely to prefer a reasonable colleague, English teacher, principal, mother, father, and partner than they were a reasonable captain, CEO, math teacher, and family doctor, *B* = −1.92, *SE* = 0.09, *z* = 21.94, *p* < .001. Similar results emerged when examining necessity scores for each role (bottom of Figure 3).

Participants considered both rational and reasonable traits as necessary for each of the roles—i.e., necessity scores were on average above “moderate amount” (3), *B* = 1.35, *SE* = 0.05, *t*(2669) = 25.06, *p* < .001. Furthermore, we observed a significant role × trait type interaction, *F*(10, 5943) = 71.94, *p* < .001. Rational (vs. reasonable) traits were perceived as significantly more necessary for CEOs, army captains. Furthermore, math teachers, family doctors, and investment bankers, 7.08 < *Z*s ≤ 11.69, *p*s ≤ .001, whereas reasonable (vs. rational) traits were perceived as significantly more necessary for a school principal, English teacher, father, mother, and romantic partner, 4.13 < *Z*s ≤ 10.14, *p*s ≤ .001. Notably, in the preregistration we had classified a family doctor as more oriented to requiring holistic considerations of subjective nuance, whereas Study 2 participants consistently viewed this role as relatively more logical and rule-based (see Figure S6; also see online supplement for additional analyses concerning characteristics attributed to family doctors)^8^. Therefore, we treat family doctor as rule-based in reported analyses. The overall models remained virtually unchanged when we classified family doctor as a holistic role, dichotomous choice: *B* = −1.62, *SE* = 0.09, *z* = −18.63, *p* < .001, continuous “necessity” scores, *B* = −0.92, *SE* = 0.04, *t* = −21.91, *p* < .001. In other words, participants preferred a reasonable agent for social roles requiring holistic, context-specific interpretation, but a rational agent for social roles requiring context-free processing and the application of logic.

Next, we assessed participant’s appraisals of each social role along a range of algorithmic (algorithmic processing, logic, agentic pragmatism) and interpersonal (warmth, closeness, and interaction frequency) characteristics. Following preregistration, we examined the extent to which preference for rational versus reasonable agents for different social roles can be explained by the difference in these role appraisals. Indirect effects analysis showed that the relative difference in attribution of algorithmic vs. interpersonal characteristics accounted for 28% [24%, 34%] of the variance in the relationship between the group (rule-based vs. not) and agent preference.

Overall, Study 2 showed systematic variability in preferences for rational and reasonable agents across various social roles requiring sound judgment. For the roles commonly appraised through the lens of analytic logic and algorithmic rules, rational agents were uniformly preferred. Conversely, for the roles commonly appraised through the lens of interpersonally sensitive, holistic characteristics, reasonable agents were uniformly preferred. It appears, laypersons do not consider the standards of rationality and reasonableness to be interchangeable and they prioritize each of these standards in some contexts but not others.

## STUDY 3

Studies 1–2 showed that laypeople recognize two deliberate standards—rationality and reasonableness—and that their spontaneous role assignments differ across algorithmic and relational dimensions (e.g., CEOs vs. parents). What remains unclear is whether activating a goal to “be rational” or “be reasonable” can causally shift whom people favor when the stakes are high.

In the pre-registered Study 3 we tested this question. Participants acted as hiring managers for five consequential sectors: law, education, healthcare, civic planning, and the military. In the *confirmatory phase*, each hiring decision began with an explicit brief: appoint the rational candidate or appoint the reasonable candidate. Candidates were matched in social characteristics and described either as adhering to a *rule-based* (stereotypically rational) or a *holistic* (stereotypically reasonable) approach to their judgment. This approached aimed to isolate the effect of motivational goal-framing on choice.

In an *exploratory phase* we held the candidate labels (rational or reasonable) constant and instead varied the decision ecology: some scenarios were framed as well-defined, others as ill-defined. We recorded comparative perceptions of trustworthiness in a binary choice between the two candidates. Hereby, we explored whether there is a heightened preference for a reasonable approach in ill-defined contexts and a rational approach in well-defined contexts.

### Methods

#### Participants.

Prolific participants completed the study on the Qualtrics platform. Participants were offered £2 to complete the 20-minute study. To be eligible for the study, participants were required to be 18 years of age or older, be fluent in English, and reside in one of the following countries: US, UK, or Canada. Pre-registered power analyses (G*Power 3.1.9.2 with *α* = 5% / *β* = 10% [due to novelty of the paradigm] and Probability H_0_ = 50%) for a logistic regression with a binomial dependent variable with a medium effect size in social psychology, *r* = .17 / OR = 1.87 (Sommet et al., 2023), we estimated a sample of 447 as sufficient. Per pre-registered plan, we round up the sample to at least 500 to account for possible attrition due to inattentive participants, expecting lower attrition from Prolific compared to the customary attrition of 30–50% from the MTurk platform (Aguinis et al., 2021). Given the repeated measures nature of the overall design, we estimated this sample to be more than sufficient to detect overall hypothesized effect across ten scenarios in the confirmatory part and eight scenarios in the exploratory part.

We recruited 509 participants. In line with the pre-registration, we excluded 7 participants due to inadequate open-ended responses. Further, we excluded scenario-specific responses where participants spent less than 15 seconds and omitted participants who were faster than 15 seconds in at least 7 out of 10 scenarios, indicating insufficient engagement. The final sample included 464 participants (Table 2).

#### Procedure.

The study comprised confirmatory and exploratory parts. In the confirmatory phase, participants took a role of a decision-maker faced with a task of selecting the most suitable candidate for a range of professional roles: lawyers, teachers, doctors, city council worker, military commander (block order randomized). Participants were either informed that their goal is to hire someone who is rational or someone who is reasonable (within-person randomized). Participants selected one of two candidates for a job (presentation randomized). For each selection task, the “rule-based” candidate was presented as favoring a step-by-step (top-down) logic, and consistently following the rules without exceptions to maximize desired preferences/outcomes. Conversely, the “holistic” candidate employed narratives and analogies in their decision-making, showed a flexible approach to rules, and was context-sensitive in their consideration of preferences and norms. While being mindful of ensuring diversity of scenarios and naturalistic description of the candidates for each role (avoiding formulaic repetitions; see online supplement for verbatim items), qualifications of candidates in each scenario were presented in a positive light, highlighting their unique strengths, but also noting relevant trade-offs that accompany these strengths. For the analytic/rule-based candidate, these competencies involved cross-situational consistency, systematicity, and reliance on hard data instead of being swayed by situational ‘noise.’ For the holistic candidate, these competencies involved use of case-specific narratives and seeking perspectives from varied stakeholders^9^.

To ensure the unique scenarios do not determine the candidate choice in the rational goal vs. reasonable goal conditions, we designed two scenarios per block reflecting a particular professional context, counter-balancing the type of scenario matched with each (rational/reasonable) goal (see online supplement for verbatim instructions for each scenario). For instance, in a legal scenario participants envisioned being a CEO of a pharmaceutics company which was at risk of being sued. As a CEO, their goal was to choose either a rational or reasonable lawyer to represent the case, selecting between two candidates. Both candidates were described as hard-working and knowledgeable. The “rule-based” candidate was described as somebody who “sticks to same set of legal rules for every case, uses step-by-step logic to argue, looks for facts that are simple and can boost the company’s chances of winning, aims to maximize company’s gains, main goal is to win the case and protect company’s money and reputation”, whereas the “holistic” candidate was described as somebody who “adjusts the rules to fit each case’s details, uses stories and examples to make points, explains how facts fit together, shows how everything is connected while considering public opinion and fairness, aims to balance what’s good for the company and what’s socially responsible.”

In the exploratory phase, we featured eight scenarios analogous to those in the confirmatory phase. We omitted the military scenarios, because typical military context calls for well-defined objectives. For each scenario, we provided an additional clarification about the nature of the job. Four scenarios highlighted the *ill-defined* context of the scenario (e.g., complex task with uncertainty and unknowns; requirement to balance different interests) and four other scenarios highlighted the *well-defined* context (e.g., clearly defined task requiring precision and logic; requirement of pursing the most favorable solution/bottom-line). For example, one healthcare scenario involved choosing a physician for a hospital serving a multifaceted community of patients at the junction of three different neighbourhoods (young and old, rich and poor), a job that involves “balancing cultural, economic, and age-related needs of patients” [ill-defined context], whereas another scenario involved choosing a doctor for a clearly defined task of long-term treatment of diseases, “involving the use of logic to estimate the most effective treatment, cost, and benefits” [well-defined context].

For each scenario, participants were prompted to compare candidates taking a *rational* and a *reasonable* approach (counter-balancing the order of a candidate presented first). Specifically, participants selected whether a rational or reasonable candidate is more trustworthy (“whom would you trust more to do a good job”), along with a few other positive characteristics (“has natural abilities that can’t be taught;” “more likely to grow with experience in this position;” “more authentic in their approach;” see verbatim instructions and analyses of these characteristics in the online supplement).

#### Materials.

In the confirmatory part, for each scenario participants selected between two candidates. In the exploratory part, participants were prompted to select which of the two candidates is more likely to show assessed characteristics. Participants were then presented with an open-ended attention check item. Finally, participants provided some demographic information concerning age, sex, country of residence, income and education.

#### Analytical Methods.

Our primary focus was on determining the influence of reasonable/rational motives on preferences for holistic candidate vs. rule-based candidate (coded as 1 and 0, respectively). We also explored preferences for a reasonable candidate (coded as 1) versus a rational candidate (coded 0) when assessing perceived trustworthiness, authenticity, natural abilities, and growth potential in the context of ill- vs. well-defined tasks. Given the binary nature of responses for confirmatory and exploratory analyses, we relied on Generalized Mixed Models with a binomial distribution, employing the *lme4* package in *R* for model fitting, with a random intercept accounting for interdependence of responses nested in participants. Furthermore, we used the *emmeans* package (Lenth, 2023) for obtaining estimated marginal means of the probability to select a holistic candidate (back-transformed from logit scores used for the binomial models), with normal approximation for the test statistics. For confirmatory analyses, we tested planned contrasts comparing mean probability for holistic (vs. rule-based) candidate in the reasonable vs. rational conditions. For exploratory analyses, we compared mean probability for reasonable (vs. rational) candidate in the ill-defined vs. well-defined context.

### Results

**Figure 4.**
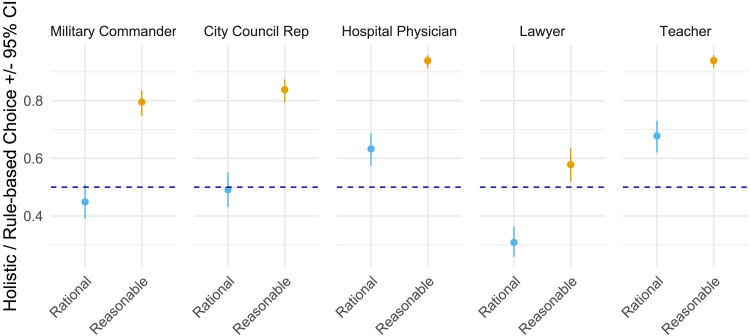
Proportions of holistic (vs. rule-based) candidates chosen for jobs when conditions call for rational and reasonable persons, for each of the five scenarios. In each scenario, participants reported significantly greater preference for a candidate following a holistic approach to judgment when the motive was to hire a reasonable compared to rational person. Proportions and 95% confidence intervals obtained via estimated means, back-transforming logits from pre-registered generalized mixed models into proportions. Scores above the dashed parity line (.05) show a preference for the holistic candidate; scores below favour the rule-based candidate.

Participants favored holistic (vs. rule-based) candidates when the goal was to hire a reasonable over a rational person (see Figure 4), *Z* = 18.75, *p* < .001, *OR* = 5.53. This effect was systematic across all scenarios, 6.91 < *Z*s ≤ 9.91, *p*s < .001; 3.08 < *OR*s ≤ 8.85.

We also explored the absolute preferences for candidates showing holistic and rule-based judgment, as indicated by the non-overlap of the 95% confidence interval with the horizontal parity line (proportion of .50; Figure 4). When the goal was to hire a reasonable person, in each scenario participants showed a significant preference for a holistic candidate. Conversely, when the goal was to hire a rational person, participants were significantly more likely to favor a rule-based lawyer and tended to favor a rule-based military commander and a rule-based city council representative, though the latter two were not significantly different from parity.

Next, we explored preferences for reasonable and rational candidates in the ill- and well-defined contexts. Participants were significantly more likely to select the reasonable (vs. rational) candidate as more trustworthy in the ill-defined context, *M*(probability) = .593, *SE* = .019, 95% CI [.556, .630] above the parity value of .50. Conversely, participants were more likely to select the rational (vs. reasonable) candidate in the well-defined context, *M*(probability) = .444, *SE* = .020, 95% CI [.406, .483] falling below parity. The contrast between trust preferences in ill- and well-defined contexts was significant, *Z* = 7.72, *p* < .001, *OR* = 1.83.

**Figure 5.**
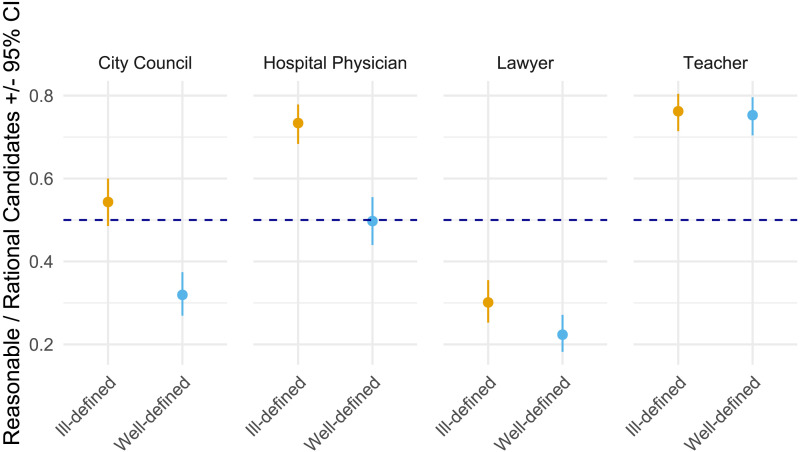
Estimated proportion of participants (marginal means ± 95% CI) who judged the reasonable candidate as more trustworthy than the rational candidate, plotted for ill-defined and well-defined versions of four roles (physician, teacher, city-council representative, lawyer). The dashed line at 0.50 marks indifference: points (with error-bars) above the line indicate an absolute preference for the reasonable candidate; points below it indicate an absolute preference for the rational candidate. Our main test concerned the *relative* context shift—i.e., greater trust in the reasonable candidate under ill-defined than well-defined conditions, which was significant for physician, city-council representative, and lawyer, but not for teacher. Looking at absolute preferences, participants trusted the reasonable teacher in both contexts (*CI*s entirely above 0.50), whereas they trusted the rational lawyer in both contexts (*CI*s entirely below 0.50), echoing the role-specific pattern found in Study 2.

Further planned contrasts revealed systematic effects of context-type in three out of four scenarios. In city council, doctor, and lawyer scenarios, participants were significantly more likely to select the reasonable (vs. rational) candidate as more trustworthy in the ill- vs. well-defined contexts, *Z* = 6.17, *p* < .001, *OR* = 2.54; *Z* = 6.76, *p* < .001, *OR* = 2.79; *Z* = 2.50, *p* = .013, *OR* = 1.50, respectively. However, for the teacher scenario, this difference was not significant, *Z* = 0.32, *ns*, *OR* = 1.05, indicating a similarly high preference for the reasonable (vs. rational) candidate in both contexts (see Figure 5).

## STUDY 4

Thus far, our experiments have demonstrated that rational and reasonable motives align differently with preferences for rule-based versus holistic judgment. Additionally, we have explored how role-context (well-defined vs. ill-defined) influences the perceived trustworthiness of individuals with stereotypically rational versus reasonable characteristics. Building on these insights, Study 4 ventured further in two directions. First, it extended the exploration of analytic rationality versus holistic reasonableness beyond the social context of judgment. Second, it aimed to directly assess whether rule-based cognitive strategies are predominantly associated with rational judgment, and holistic strategies with reasonable judgment. We hypothesized that asking participants how a *rational person* would categorize would steer them toward a rule-based solution that hinges on a single explicit diagnostic feature, thereby down-weighting broader pattern-based similarities. Conversely, when asked how a *reasonable person* would categorize, we expected a preference for holistic grouping that integrates multiple incomplete similarities, even at the expense of parsimony. To test this hypothesis, participants were asked to categorize objects according to holistic and rule-based principles from a perspective of rational and reasonable persons.

### Methods

#### Participants.

MTurk participants completed the study on the Qualtrics platform. We offered participants USD $1.00 to complete the 10-minute survey. To be eligible for participation, we recruited individuals to be at least 18 years of age, reside in one of Canada or U.S. and possess a HIT approval rating at or above 90% (see Table 2). Following pre-registration, we excluded participants who had an IP address that was either a duplicate to an IP address already collected or located outside of North America, completed the entire survey in under 370 seconds, incorrectly answered or did not provide an answer to the comprehension question of the practice trials, provided a nonsensical answer to the open-ended question of the study, as well as indicated that we should not use their data for reasons of them being tired, distracted, or them not reading the instructions carefully. The final sample consisted of 411 participants. This surpassed our minimum sampling goal specified in our preregistered power analysis; we powered for effect size *r* = .20. Per G*Power 3.1.9.2, with alpha and beta each set to 5%, we would require 314 to conduct a two-tailed test.

#### Procedure.

**Figure 6.**
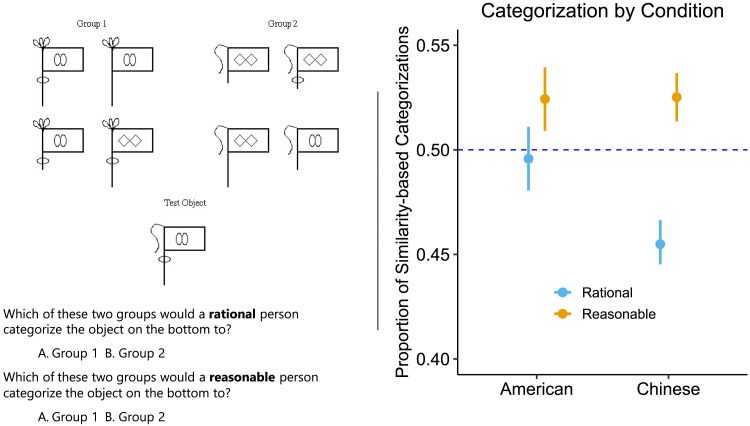
Example trial from the categorization task (left panel) and the estimated marginal means (proportions) from a generalized linear mixed model of similarity-based categorizations made for each condition across the 20 trials of the American (Study 4) and Chinese (Study 5) samples (right panel). In the left panel example trial, categorizing the stimulus as Group 1 as a holistic similarity judgment, whereas Group 2 as a rule-based judgment (as each object shares a common feature: the curved line extending from the top left of the rectangle). In the right panel graph, the estimated marginal means of each condition are represented via a dot with surrounding 95% within-subjects confidence intervals.

To begin, we provided participants with descriptions of two methods to categorize objects (see Figure 6, left). One method was said to be based on “overall similarity” between the stimulus to be categorized and the members of a particular category. Using this method, a stimulus would be categorized into Group 1 instead of Group 2 if the stimulus appeared to be more similar to the members of Group 1 than the members of Group 2. The other method was introduced as the “single feature categorization method”—i.e., categorizing depending on only one feature. Using this method, a stimulus would be categorized into Group 2 instead of Group 1 if the stimulus possessed a unique feature that all the members of Group 2 possessed and none of the members of Group 1 possessed.

We first checked participant’s comprehension of the difference between these categorization methods by asking participants to indicate for the same picture which method refers to overall similarity (i.e., selecting a group that is similar even if none of the objects share a single feature; Group 1 in Figure 6) or a rule-based judgment (i.e., selecting a group where each object shares a common feature; Group 2’ curved line in Figure 6). If participants chose the incorrect option in the comprehension check, the survey provided a prompt explaining the definition of the respective method. Thus, we ensured all participants had a clear understanding of the difference between the two approaches to object categorization before they then reported what types of categorizations rational and reasonable people would make for each stimulus.

Next, we asked participants to consider how different people (reasonable people and rational people) would categorize a set of test objects. Across 20 trials, participants made two categorization judgments: one based on which set a *rational* person would think the object most similar to and one based on which set a *reasonable* person would think the object most similar to (see left panel in Figure 6 for verbatim questions). The presentation order of the trials was randomized, and the presentation order of the reasonable and rational questions was counterbalanced across participants. After completing the categorization task, participants recalled and explained what they did during the task. To conclude the survey participants provided some demographic information.

### Results

We tested whether people use a rule-based strategy when instructed to categorize items like a rational person and use a more holistic strategy when instructed to categorize items like a reasonable person. If people view the rational standard as placing greater emphasis on the application of specific, reductive rules than the reasonable standard and view the reasonable standard as placing greater emphasis on consideration of the overall context than the rational standard, then we should expect more holistic (and thus, fewer rule-based) categorizations when judging for a reasonable person compared to a rational person.

The results supported our hypothesis. Using a generalized linear mixed model (binomial distribution of forced-choice categorizations; 0 = similarity based, 1 = rule-based), with participants nested and condition and trial as mean-centered predictors, we found that participants made significantly more similarity-based categorizations when judging for a reasonable person than for a rational person, *B* = 0.12, *SE* = 0.03, *z* = 3.59, *p* < .001. Though the effect size of the observed difference was small, *d* = .12, 95% CI [.053, .181], it may be practically meaningful when accumulated across many situations inviting people to make such a choice in their lives (Funder & Ozer, 2019). Results were comparable when estimating effects for the first trial, only, *B* = 0.13, *SE* = 0.06, *z* = 2.13, *p* = .033. Furthermore, the results did not significantly differ when trial was omitted from the model. In addition, as Figure 6 illustrates, participants made significantly more similarity-based categorizations when judging for a reasonable agent than one would expect from chance, *Z* = 2.28, *p* = .022, *d* = 0.11, 95% CI [0.01, 0.21]. Conversely, participants favored a rule-based categorizations when judging for a rational agent (Figure 6), though this preference was not significantly different from chance, *Z* = −0.52, *p* = .606, *d* = 0.03.

Overall, Study 4 showed that lay understanding of rationality and reasonableness varies in the degree to which each emphasizes holistic processing and demonstrated that these folk theories generalize to non-social cognitive judgments. When asked to choose like a reasonable person, participants were more likely to select a pattern matching overall family resemblance to the target stimuli, even if no single feature was shared by each item in the set. In contrast, when asked to choose like a rational person, participants tended to pick a pattern matching a single feature shared by each item in the set, even if the pattern overall was more different from the target. These results are consistent with the notion that people prioritize different standards of sound judgment depending on the situation. We note, however, that the categorization preference for a rational person was not significantly different from parity, inviting a replication in Study 5.

## STUDY 5

Inferences from prior studies are limited to English-speaking North America and Western Europe. It is an open question whether the coexistence of rational and reasonable standards generalizes across cultures varying in values, linguistic semantics, and socio-economic history. If these standards facilitate navigation of distinct but frequently encountered situations (those with well-defined parameters versus those with unknown or intractable elements), similar patterns might emerge globally, as both situation types are common in many societies. However, if the rational-reasonable lay distinction is rooted in cultural environments influenced by European Enlightenment ideals or shaped by neo-liberal institutions (e.g., Pinker, 2018), it may be less pronounced in non-Western cultures, such as Chinese society, which has a distinct socio-economic history and a different value system. To this end, we examined Chinese participants’ expected contributions from rational and reasonable persons in a Dictator Game, along with characteristics they ascribe to rational and reasonable persons in Mandarin.

Second, we aimed to replicate and extend the cross-over effects related to rational and reasonable standards in non-social judgment. Specifically, we examined whether the stronger association of a reasonable standard with holistic judgment, as opposed to the weaker link of a rational standard with rule-based judgment (Study 4), represents a consistent finding or an anomaly. Third, in Study 5 we aimed to simultaneously test the impact of rational and reasonable standards for economic and non-social judgments, as well as for spontaneous trait inferences, within the same research design. This approach enabled us to explore cross-domain consistency in differentiating between rational and reasonable standards across economic and non-social judgments.

### Methods

We sought to test folk standards of good judgment by examining general perceptions of rational and reasonable agents (similar to Study 1; also see Grossmann et al., 2020; Study 1), expected contributions by rational and reasonable agents in a Dictator Game (replication of Grossmann et al., 2020; Study 5, on a Chinese sample), and differences in the categorization of non-social objects (replication of Study 4).

#### Participants.

Participants were recruited via Sojump, a Chinese online survey platform from a pool of over 2.6 million active users and completed the survey on the same platform. Participants were offered CNY ¥2.50 to complete the 10-minute survey. Participants were required to be at least 18 years of age and reside in mainland China. Participation was restricted to Mandarin speakers. We recruited a sample of 748 participants after excluding a further 49 participants following preregistered criteria. This surpassed our minimum sampling goal specified in our a priori power analysis; we powered for effect size *r* = .20. Per G*Power 3.1.9.2, with *α* and *β* each set to 5%, we would require 314 participants per presentation order (of which there were two) to conduct a two-tailed test.

Sojump excluded 2,159 potential respondents based on a combination of their internal quality standards and specifications set by the research team (not possessing at least a high-school education, failing to take the experiment seriously). The data of participants excluded was not made available to the researchers. The final sample of 697 participants was younger than samples in Studies 1–4 (*M*_age_ = 27.24, *SD*_age_ = 6.94), had somewhat more females than males (60% female), was ethnically homogenic (100% Chinese) and had completed on average 11.83 (*SD* = 3.15) years of education after primary school.

#### Procedure.

The study began with a Dictator Game (Kahneman et al., 1986). Participants were asked to indicate how much of the 100 units of currency they would offer if they were Player A as well as how much they thought a rational person and a reasonable person would offer if they were each in the role of Player A (see supplementary Study 5 Materials for details). Next, participants completed a modified version of the adjective task implemented in Study 1 (three adjectives describing a person who shows reasonable judgment and a person who shows rational judgment; in counterbalanced order). Finally, participants completed the categorization task (see Study 4 Methods for details) and provided demographic information.

#### Materials.

All materials were backtranslated to ensure semantic equivalence in English and Chinese. Further, to establish more accurate terms for ‘rational’ and ‘reasonable,’ we examined local definitions in standard textbooks and consulted local experts in psychology and economics, cross-validating the chosen terms by inspecting the suggested terms in Google Translate.

#### Coding of Adjectives.

We followed the classification in Grossmann et al. (2020), sorting back-translated versions of the top 25 most frequent adjectives in terms of V1 (analytic vs. interpersonal) and V2 (context independent vs. socially conscious) themes (see Chinese and English version on OSF; https://osf.io/us637). For V1, ‘analytic’ involved computational, analytical, or logical characteristics, while ‘interpersonal’ relates to attitudes or relationships between people. For V2, ‘context independent’ refers to broadly applicable or individual-focused traits, and ‘socially conscious’ pertains to concern for others, social norms, or prosociality. For more detailed information, refer to the online supplement.

### Results

We first tested expectations for rational and reasonable agents in a Dictator Game, which pits maximizing self-interest against fairness to another person. Consistent with prior North American findings (Grossmann et al., 2020; Study 5), out of 100 units of currency, participants expected reasonable agents (*M* = 46.13, *SD* = 11.34) to contribute more than rational agents (*M* = 29.02, *SD* = 20.05), *t*(697) = 22.37, *p* < .001, *d* = 0.85, 95% CI [0.76, 0.93], with personal choice (*M* = 35.12, *SD* = 19.85) in-between reasonable, *t*(697) = 17.17, *p* < .001, *d* = 0.65, 95% CI [0.57, 0.73], and rational agents, *t*(697) = 9.30, *p* < .001, *d* = 0.35, 95% CI [0.28, 0.43].

We next examined participants’ folk theories of rationality and reasonableness, examining types of adjectives participants spontaneously generated when prompted to characterize a person who shows rational judgment and a person who shows reasonable judgment (see Table 3 for top 10 more frequent adjectives). Like in Study 1, we first examined if people were more likely to mention intuitive characteristics when describing reasonable rather than rational persons: Participants did not mention characteristics that would chiefly rely on intuition either among the top 10 (Table 3) or top 25 characteristics (Table S8 in the online supplement), suggesting little difference in emphasis on intuition in spontaneous descriptions of the rational and reasonable persons in this sample.

**Table 3.** Most frequent listed adjectives (top 10) describing rational and reasonable persons in China.

**Rational** (理性的)			**Reasonable** (通情达理的)		
Word (English)	Word (Chinese)	Frequency (%)	Word (English)	Word (Chinese)	Frequency (%)
calm	冷静	11.0	kind/kind-hearted	善良	10.0
intelligent/smart	聪明	8.1	intelligent/smart	聪明	3.7
reason	理智	5.2	tolerant	宽容	2.5
wise	睿智	4.4	empathetic	善解人意	2.4
rigorous	严谨	2.5	rational	理性	2.1
cautious	谨慎	2.0	sensibility	感性	2.1
wisdom	智慧	2.0	fair	公平	2.0
steady	稳重	1.5	magnanimous	大度	1.9
logic	逻辑	1.5	generous	大方	1.8

This table presents the top 10 most frequently listed adjectives used by Chinese participants to describe rational (理性的) and reasonable (通情达理的) individuals. The adjectives are provided in English along with their corresponding Chinese characters, and the frequency of each adjective is expressed as a percentage of the total responses. English terms correspond to Google Translate suggestions for simplified Chinese and were cross-checked with the bilingual co-author.

Next, we explored whether participants conceptualized rational agents as analytical and emphasizing individualistic characteristics and reasonable agents as holistic and emphasizing the integration of social norms. Coding the top 25 most frequent listed adjectives as either algorithmic or interpersonal and computing a 2 × 2 contingency test revealed that, proportionally, participants used significantly more analytic words compared to interpersonal words to describe rational persons compared to reasonable persons, *χ*^2^(1) = 447.98, *p* < .001, Φ = 0.45, 95% CI [0.41, 0.49]. In addition, coding the 25 most frequent listed adjectives as either socially conscious or context independent and computing a similar 2 × 2 contingency test revealed that the frequency of socially conscious words spontaneously generated was far greater for reasonable agents than rational agents, *χ*^2^(1) = 360.21, *p* < .001, Φ = 0.40, 95% CI [0.36, 0.44].

Consistent with earlier work on North American samples (Grossmann et al., 2020; Study 1), Chinese participants described rational persons using terms more narrowly focused on the qualities of logic and reason. Conversely, they described reasonable persons through a combination of some analytical terms similar to those used to describe rational persons, such as “intelligent/smart,” but also using unique civic virtues such as sensibility, tolerance, and magnanimity (indicating the reasonableness standard is more holistic than the rational standard). Further, we found similarities and differences in the attributions for rational compared to reasonable agents. Concepts of rationality and reasonableness overlapped in their focus on cognitive competences (intelligence and/or smart). However, rational agents were also described using terms focused on the qualities of logic and reason, while reasonable agents in terms related to civic virtues such as sensibility, tolerance, and magnanimity.

Using a generalized linear mixed model, we next conducted a logistic regression of the categorization data. Participants made significantly more similarity-based categorizations when judging for a reasonable person than for a rational person, *B* = 0.27, *SE* = 0.02, *z* = 10.95, *p* < .001 (Figure 6), with a moderate effect size, *d* = .27, 95% CI [.224, .321]. Results were comparable when estimating effects for the first trial, only, *B* = 0.18, *SE* = 0.05, *z* = 3.71, *p* < .001. Furthermore, participants made significantly more similarity-based categorizations when judging for a reasonable agent than one would expect from chance alone, *Z* = 5.21, *p* < .001, *d* = 0.14, 95% CI [0.07, 0.22]. Likewise, participants made significantly more rule-based categorizations when judging for a rational agent than one would expect from chance alone, *Z* = 9.03, *p* < .001, *d* = 0.46, 95% CI [0.12, 0.27]; see online supplement for the formal comparison of results in Studies 4–5.

Overall, Study 5 provided evidence that lay conceptions of two standards of sound judgment, rationality and reasonableness, appear similar in the Chinese context to the North American context of prior work, with systematic differences in expected contributions in a Dictator game, spontaneously generated list of traits for rational and reasonable persons, as well as categorization of non-social objects.

## GENERAL DISCUSSION

We proposed that lay people recognize at least two distinct standards for how to deliberate to reach sound judgment which in the English language are conventionally labelled rationality and reasonableness (also see Rawls, 1999). Lay rationality, in the context of our framework outlined in Table 1, is primarily understood as a folk standard involving analytic, rule-based, and reductive reasoning. It is a process characterized by a systematic and logical progression from well-defined premises to clear conclusions. This lay standard of deliberate judgment is particularly applicable to well-defined problems, where all relevant variables are known and fixed, and clear rules are in place for integrating these variables. Lay rationality, therefore, tends to focus on the maximization of the most immediately apparent form of utility, namely the one involving self-interest, aligning with a hierarchical structure of values, and often seeks to reduce complex scenarios to a single dimension of valuation.

In contrast, lay reasonableness is viewed as a more context-sensitive, case-based, and holistic approach to judgment. It moves beyond the linear and mechanical process of rationality, embracing dialogic reasoning that incorporates social conventions, norms, and rhetorical strategies, such as reasoning by analogy and narratives. Lay reasonableness is particularly applicable to ill-defined problems, where relevant variables are uncertain, unknown, or lack clear rules for integration. This approach balances incommensurable values that cannot be reduced to a single dimension of valuation, emphasizing a horizontal or interconnected network of values and demonstrating a higher sensitivity to context.

In five studies we provide support for this theoretical distinction between lay concepts of rationality and reasonableness in the context of sound judgment, following a set of hypotheses we outlined in Table 1. First, addressing our initial hypothesis across spontaneous trait listings, semantic networks, and factor analyses (Studies 1a–1b), laypeople treated rationality and reasonableness as two deliberative yet qualitatively different ideals of sound judgment; the same psycholinguistic split reappeared in free-response Mandarin narratives (Study 5). Second, addressing our hypothesis concerning context-sensitive preferences, when choosing agents for everyday roles (Study 2) or for high stakes hires framed as well-versus ill-defined (Study 3), participants shifted toward rationality in well-defined situations and toward reasonableness when problems were ill-defined. Third, addressing the hypothesis about the generalizability of the distinction between lay rationality and reasonableness to non-social cognition, in two categorisation tasks (Studies 4–5) a “rational” chooser was expected to apply reductionist rules, whereas a “reasonable” chooser was expected to group holistically. These patterns replicated across English and Mandarin speakers from North America, the UK, and China, underscoring the cross-cultural reach of these folk theories.

This research greatly expands on observations from economic decision-making, which suggest that laypeople associate rationality with self-interest and reasonableness with fairness (both in between- and within-subject experiments; Grossmann et al., 2020). Building on this work, here we showed that in the context of sound judgment lay rationality and reasonableness are both associated with chiefly deliberative qualities and possess distinct profiles of judgmental attributes. We also demonstrate how people assign distinct value to rational or reasonable choice across different contexts and apply these folk standards of judgment to non-social domains. These findings provide valuable evidence for a new theoretical model of meta-judgment (Grossmann & Eibach, 2024), simultaneously raising questions about other judgmental standards beyond rationality and reasonableness. Scientific models of judgment and decision-making, such as the somatic marker hypothesis (Damasio, 1996; Poppa & Bechara, 2018) and the dual systems model (Kahneman & Frederick, 2002), increasingly recognize that people employ diverse types of deliberation in everyday judgment and decision-making (e.g., De Neys, 2021; Evans & Stanovich, 2013; Melnikoff & Bargh, 2018). These models also assume that laypeople are metacognitively aware of these heterogeneous types of judgment and apply this knowledge to either select a particular type of judgment or integrate multiple types of judgment to guide their decision-making in particular situations (e.g., Grossmann, 2017; Loewenstein et al., 2001; Petracca, 2021). This highlights the importance of examining whether laypeople’s metacognitive beliefs distinguish between types of judgment and recognize their relevance to different functional contexts, as the present studies do by investigating metacognitive awareness of the rational-reasonable distinction.

The insight that folk standards of sound judgment vary across situations dovetails with other research in cognitive, differential, and social psychology. In particular, work in moral psychology makes an analogous distinction between *instrumental rationality* (endorsing sacrificial harm to maximise aggregate welfare) and *impartial beneficence* (treating everyone’s welfare equally), observing the reputational cost of ‘cold’ instrumental rationality in relational roles (Everett et al., 2018). Specifically, observers strongly prefer the non-instrumental agent as a friend or spouse, yet that advantage shrank or disappeared for a boss or political leader when the dilemma concerned purely impartial allocation. This observation is consistent with the preference for reasonable versus rational agents for relational roles observed in our Studies 2–3. This role sensitivity also accords with the ‘argumentative theory’ of human reasoning: reasoning evolved chiefly as a social tool for persuading and coordinating with others rather than as an asocial optimiser, so it naturally toggles between analytic argument construction and audience-oriented norm sensitivity (Mercier, 2016). Complementary to these relational and decision-ecology perspectives, reasonableness may be valued because it fosters perspective learning: by engaging multiple stakeholders, the reasoner acquires insights into others’ idiosyncratic goals—just as intuition can uncover one’s own authentic preferences (Oktar & Lombrozo, 2022; Varga & Guignon, 2020).

Moreover, the concepts of maximizing and satisficing (Schwartz et al., 2002) map onto the underlying value structures of rationality (maximize a single value) and reasonableness (ensuring all relevant values are satisfied to a minimum extent). Likewise, a sound decision could be framed as one that achieves one’s goals efficiently independent of justification (Evans & Over, 1997), the definition of which necessarily applies to contexts where one has a single goal and to contexts where one has multiple competing goals. Further, scholars studying group dynamics claim that a good decision adheres to local notions of morality (Kessler & Cohrs, 2008) and that a person’s commitments may require one to act against one’s self-interest broadly construed (Sen, 1997). Alternatively, good judgment might serve to elevate an identity role important to the agent (Hirsh & Kang, 2016), the process of which would vary across identities and contexts. Sound judgment might also be construed as being free of common cognitive biases (Carnevale et al., 2011), yet knowing if one is truly unbiased is no easy task (Horwich, 1987) and unlikely to be a necessary feature of sound judgment (i.e., reasonableness) despite probably being a sufficient one (i.e., rationality). This non-exhaustive highlighting of relevant literatures suggests that scholars, in their attempts to construct prescriptive models of good judgment, may have effectively captured two central tenets of folk theories of judgment.

Reasonableness also resonates with Rawls’ (1999) method of reflective equilibrium, in which agents seek a “good enough” fit between general principles and concrete cases through an iterative, internal explanatory dialogue where the decision-maker simulates a process of pitching reasons for a decision option to an imagined neutral party similar to Mead’s “generalized other” (Mead, 1934). The process continues until the proposed reasons for a given option reach a threshold of acceptability for the simulated third party or until a time constraint is passed. At that point the decision-maker chooses whatever option came closest to meeting the neutral party’s acceptance threshold. Psychologically, this process looks less like maximizing a single utility function and more like an internally simulated process of dyadic bargaining—i.e., a check that no salient stakeholder or norm is egregiously violated (Knight, 2025; Simon, 1956). Framing lay reasonableness in these terms underscores why it need not collapse into group-level utility maximization yet still admits formal treatment.

The present findings offer insight for diverse areas of scholarship, each exploring human judgment, such as experimental jurisprudence, cognitive science, and decision sciences. Experimental jurisprudence researchers (e.g., Sommers, 2021) may draw from this work a more complete framework as to how people understand and apply the legal concept of reasonableness. Cognitive scientists may wish to explore the ramifications for these two standards beyond categorization tasks, especially in contexts where rule-based reasoning competes with prior beliefs about the world (e.g., belief bias and syllogistic reasoning; Evans et al., 1983). The decision sciences, especially computational ones, ought to consider the interplay between the two standards in a given moment and over time; according to laypeople good judgment is not always exclusively rational nor reasonable. For instance, as an individual gains contextual knowledge and a better understanding of the relevant parameters a preference for reasonable judgment may switch to rational judgment. Alternatively, in contexts where the individual prefers rational judgment but has their knowledge of the relevant parameters undermined (i.e., through a Black Swan event; Taleb, 2007), reasonable judgment may be sounder. Such variation in contextual affordances parallels the finding that physicians and laypeople make different decisions when evaluating an individual patient than when considering a group of comparable patients (Redelmeier & Tversky, 1990). It also dovetails with observations about preference for a rational (vs. reasonable) family doctor (Study 2; focus on individual patient and their needs) and a rational (vs. reasonable) physician to provide the best treatment to a patient (well-defined context condition in Study 3) and a preference for a reasonable (vs. rational) hospital physician for a diverse community (ill-defined context condition in Study 3).

Future studies should examine whether the present findings hold across different cultural, historical, and technological settings. In particular, researchers can test how our findings apply to artificial intelligence (AI) and other decision aids (e.g., Arkes et al., 2007). For example, people who value rationality may favor AI-based healthcare diagnostics, while those who value reasonableness may prefer human-centric approaches. Notably, research on clinical vs. actuarial judgment and algorithm aversion (Highhouse, 2008; Burton et al., 2020) shows that people often favor holistic methods even when analytical methods yield better results, suggesting they may not always recognize which approach is more effective. These insights could guide the design of AI tools that account for different judgment styles. Indeed, recent trends indicate that AI tools are developing ever-more sophisticated ways to simulate reasonable style of judgment and decision-making, which may determine the readiness of the public to apply AI tools to assist with more ill-defined problems. In turn, models of human judgment should reflect how ideas of sound judgment shift across contexts.

Future research should also probe how the rational–reasonable distinction shapes people’s vulnerability to, and correction of, mis- and disinformation. Analytic fact-checking tools and algorithmic credibility scores resonate with lay rationality: they parse claims into facts, trace sources, and output binary truth values. Yet large-scale studies show that purely analytic interventions often improve factual accuracy without shifting downstream political behavior (Nyhan & Reifler, 2019). Conversely, accuracy nudges rooted in social norms, narrative inoculation, and community deliberation may leverage lay reasonableness by embedding factual evaluation in shared values and identities (e.g., Pennycook & Rand, 2022). Understanding how citizens toggle between these two folk standards may help designers create hybrid interventions (for example, pairing real-time fact cheques with venue-specific dialogue prompts) to curb the spread of fake news without eroding trust.

A few caveats are in order before concluding. First, we relied on English and Chinese-speaking participants from post-industrial societies, which limits generalizability. Second, our work did not examine how folk standards may impact the process of making a decision and how individuals may switch between standards while making decisions, calling for further ecological validity tests. Additionally, the reliance on convenience samples may not represent less educated populations (cf. Grossmann et al., 2020). Finally, in Studies 4–5, our methodological focus on rule-based versus similarity-based strategies in assessing rational and reasonable judgments led us to include a comprehension check. This single-item comprehension check aimed to clarify judgment criteria for participants, potentially constraining the spontaneity of their alignment with rational (rule-based) and reasonable (holistic) judgments (but note similar results for the estimates of effects for the first trial, only; also see Study 3 Results showing a spontaneous preference for holistic judgment for reasonableness and rule-based judgment for rationality). Future studies should consider diverse populations and methodologies like behavioral observation, to surpass self-report and online study limits and deepen our understanding of rationality and reasonableness in decision-making.

In conclusion, our examination of rationality and reasonableness, anchored in both modern discourses and philosophical traditions, reveals the pluralist and context-dependent nature of sound judgment. Our findings indicate that laypeople systematically distinguish between different types of sound judgment, which parallels some of the philosophical discourse on this distinction between different forms of judgmental deliberation (e.g., Rawls, 1999). These findings lay the groundwork for future exploration into whether and how standards of rationality and reasonableness may continue to be used by laypeople to define sound judgment as societies cope with increasingly complex challenges, such as global decision-making about climate change, overreliance on context-free in data-driven approaches to policymaking (Fazelpour & Danks, 2021; O’Neil, 2016), and as human judgment intersects with the evolving capacities of generative artificial intelligence (Grossmann et al., 2023; Johnson et al., 2024).

## ACKNOWLEDGMENTS

We thank Mané Kara-Yakoubian for research assistance and Kerem Oktar, John V. Petrocelli, and anonymous reviewers for their thoughtful comments on the prior version of the manuscript.

## FUNDING INFORMATION

I.G. was supported by Social Sciences and Humanities Research Council of Canada Insight grant 435-2014-0685, John Templeton Foundation grant 62260, and TWCF grant TWCF-2023-32568.

## AUTHOR CONTRIBUTIONS

IG: Conceptualization; Data curation; Formal analysis; Funding acquisition; Investigation; Methodology; Project administration; Visualization; Writing – original draft; Writing – review & editing. RPE: Conceptualization; Methodology; Writing – original draft; Writing – review & editing. NK: Investigation; Methodology; Writing – review & editing. HZ: Data curation; Investigation; Methodology; Writing – review & editing. EAM: Data curation; Formal analysis; Writing – review & editing.

## DATA AVAILABILITY STATEMENT

The data, materials, and analyses scripts for each study are publicly accessible on the Open Science Framework (OSF; https://osf.io/excny/).

## Notes

^1^ Formally, many coordination problems can be translated into social-welfare terms. Inequity-averse utility functions or contractualist “virtual-bargaining” models, for instance, maximize joint pay-offs subject to fairness constraints (Fehr & Schmidt, 1999; Levine et al., 2024). Our aim here, however, is empirical: we test whether laypeople *recognize* reasonableness as a fairness-oriented standard, alongside a self-optimizing one, leaving a full formalization to future work.^2^ Normatively, the *process* of sequentially matching different features to a target object and updating evidence in light of similarity across all objects can be described as *rational* in a Bayesian sense. Thus, the Bayesian norms of rationality may be more aligned with lay understanding of reasonableness, with its focus on overall similarity, rather than lay rationality, with its focus on reductionist consistency.^3^ The switch from ‘sound’ judgment in Study 1a to ‘good’ judgment in Study 1b aimed to rule out possible misunderstanding of ‘sound’ judgment as a judgment of sounds.^4^ Supplementary topic modelling analyses of Study 1a participants’ narrative responses of the personal understanding of “sound judgment” suggested the centrality of each term to the broader notion of sound judgment, as demonstrated by their prevalence in the most dominant themes (see Figure S2). Critically, in most topic modelling solutions ‘rational’ and ‘reasonable’ reflected distinct themes, rather than parts of the same broader theme. For instance, in the 15-topic solution, which we deemed most optimal based on the balance of exclusivity and semantic coherence, ‘rational’ topic concerned an objective analysis, whereas a distinct reasonable topic concerned a context-oriented consideration and weighting of options.^5^ Because each participant could list only five adjectives chosen from an unrestricted lexicon that ultimately contained more than 400 unique terms in Studies 1a–1b, each, even the strongest pairwise co-occurrences were mathematically capped in the low-teens percentage range; thus, we focused on the relative ordering of adjectives for ‘rational’ versus ‘reasonable,’ not the absolute percentages.^6^ In a similar vein, when examining common themes in Study 1a participants’ *spontaneous* descriptions of the meaning of sound judgment, both ‘rational’ and ‘reasonable’ appeared in the top 20 words used, each mentioned by 5% of participants (across 460 narratives made of 999 unique words). Likewise, when examining Study 1b participants’ views on the meaning of good judgment in a challenging situation, ‘rational’ (20^th^ common word) and ‘reasonable’ (50^th^ common word) appeared more frequently than 90% of other words in the corpus (rational > 97% / reasonable > 94%; out of 947 unique words).^7^ In person perception, analytical agency and socially conscious communion emerge as distinct, positively interrelated, dimensions because they encompass distinct values and competences (Abele & Wojciszke, 2007). We expected that the distinction between agency and communion might be more pronounced when perceivers focus on a rational person versus a reasonable person. This is based on our hypothesis that folk theories conceptualize rationality as logic-based judgment in service of a single relevant value, which may sometimes entail the rational person employing their agency in non-communal ways.^8^ Why did participants attribute greater rationality (vs. reasonableness) to family doctors? Though speculative, our supplementary results suggest that participants perceived family doctors as advocates for and guardians of their *individual* health interests (Figures S5–S7). This perception aligns with an expectation for personalized care in privatized family medicine—one pays for a family doctor who can provide the best care for oneself and provide a sense of certainty in personally stressful situations (Kassirer et al., 2020). This perspective deviates from the bigger picture view of physicians as people who balances the needs of multiple patients simultaneously, as common in a hospital setting. We build on this speculation in Study 3, directly targeting doctors in the hospital setting.^9^ It is noteworthy that many judgmental strengths can also be perceived as weaknesses, depending on the nature of the task. For instance, cross-situational consistency in rule-following can be construed as inflexibility. In the same way, sensitivity to case-specific narratives may be construed as the insider view to judgment (Kahneman & Lovallo, 1993), which may be maladaptive when it comes at the expense of considering the baserate information.
